# Validation of a new instrument for assessing attitudes on psychedelics in the general population

**DOI:** 10.1038/s41598-022-23056-5

**Published:** 2022-10-29

**Authors:** Marija Franka Žuljević, Ivan Buljan, Mia Leskur, Mariano Kaliterna, Darko Hren, Darko Duplančić

**Affiliations:** 1 Department of Medical Humanities, School of Medicine, University of Split, Šoltanska 2, 21000 Split, Croatia; 2 Department of Research in Biomedicine and Health, School of Medicine, University of Split, Split, Croatia; 3 Department of Psychology, Faculty of Humanities and Social Sciences, University of Split, Split, Croatia; 4 School of Medicine, University of Split, Split, Croatia; 5 Department of Psychiatry, University Hospital Centre Split, Split, Croatia

**Keywords:** Medical research, Psychology

## Abstract

Although there is research interest to assess attitudes on psychedelics, no validated instrument exists for this purpose. We aimed to develop and examine the psychometric properties of the Attitudes on Psychedelics Questionnaire (APQ) in a sample of the Croatian general population. A cross-sectional, web-based survey among the general population was conducted on 1153 participants (62.1% female, 77.7% with a graduate or high school degree, 15.1% health care workers). We assessed participants’ ability to recognize psychedelic substances using a short knowledge test. The APQ consists of 20 items with four sub-scales: *Legal Use of Psychedelics*, *Effects of Psychedelics*, *Risk Assessment of Psychedelics*, and *Openness to Psychedelics*. This model demonstrated best fit in a confirmatory factor analysis. Total scale reliability was excellent (McDonald’s ω = 0.949, 95% CI = 0.944–0.953). A strong correlation with a similar unvalidated measure (r = 0.885, P < 0.001) demonstrated convergent validity. We observed an association between attitudes and knowledge on psychedelics (r = 0.494, P < 0.001). Younger age, male gender, and lower educational status were associated with higher APQ scores. The APQ is valid, reliable, and could be applied in assessing educational interventions, patients’ treatment outcomes, and the attitudes of different groups of experts. We encourage further validation of the APQ in English.

## Introduction

Psychedelics are a group of hallucinogenic psychoactive substances with action at the serotonin 2A (5-HT2A) receptor^1^. They have been controversial throughout modern history in regards to both their recreational and medical uses^2,3^. After the 1943 discovery of the psychedelic potential of lysergic acid diethylamide (LSD)^4^, psychedelics were used in psychotherapeutic research, involving an estimated number of tens of thousands of patients over a period of around 15 years^5^. Widespread and uncontrolled use and manufacture of psychedelics outside the research and therapeutic settings led to concerns about health and safety of the public. Consequently, restrictive policies were implemented leading to a 25-year hiatus of psychedelic research^3,6^. Today, most psychedelics are illegal throughout the world under UN conventions^7^. However, psychedelic-assisted psychotherapy has recently experienced renewed research interest, with strong potential implications for the treatment of different types of mental illness^8,9^.

Studies on psychedelics are increasingly demonstrating both safety and efficacy in the treatment of depression, end-of-life distress, and obsessive–compulsive disorder^2,10–12^. Psychedelics may also be effective in treating alcohol and substance abuse, as well as a smoking cessation aid^13–15^. Although it is not a classical psychedelic, MDMA is being studied as an adjunct to psychotherapy for post-traumatic stress disorder (PTSD) and is often mentioned alongside classical psychedelics due to some similarities in their effect and chemical structure^1,16,17^. Phase 3 trials of MDMA-assisted psychotherapy have demonstrated effectiveness for PTSD^18^. There is a tendency to include the recreational drug ketamine within the psychedelic group, although it is classified as a dissociative hallucinogen due to its mechanism of action via N‐methyl‐D‐aspartate (NMDA) receptor antagonism and the subjective dissociative nature of its effect^19^. Its enantiomer, esketamine, has been approved for the treatment of treatment-resistant depression^20^ and for short‐term management of suicidal thoughts^21^. Ibogaine, an alkaloid similar in structure to the classical psychedelics, has also shown potential in treating addiction to various substances such as opiates and alcohol^22^. Since 2012, the US Food and Drug Administration has given the breakthrough therapy designation to two psychedelics, MDMA and psilocybin, for PTSD and depression, respectively^23^. These developments have strengthened the public optimism surrounding psychedelics. However, some have advised caution against the extreme optimism present among psychedelic researchers and have encouraged increased transparency and replication of psychedelic research^24,25^. It is also well known that clinical trials involving psychedelics cannot fully blind their participants to the intervention arms, which may lead to overestimations of the effects sizes of psychedelic-assisted psychotherapy interventions^26,27^. The efficacy of psychedelic-assisted psychotherapy also needs to be reconfirmed in trials with larger sample sizes^10^.

In a rapidly developing research field such as that of psychedelic research, it is important to understand how the general public views psychedelics, as well as any specific sub-group, for example, professionals in the mental health field, policymakers or patients. This is in line with the recommendation in the evidence-based research approach to gain knowledge of various end-user perspectives (clinicians, patients, policymakers) on a certain issue before deciding to undertake a new study^28^. Systematically assessing and measuring attitudes on psychedelics in a variety of settings and groups could help understand the wider context and implications of their medical use for psychiatry and society in general. Any further developments in psychedelic research may also affect public opinion trends, which should be followed over time. A validated psychometric instrument allows for direct comparison and replication of data and is best suited to this purpose.

Attitudes on psychedelics have not been extensively studied. Barnett et al. assessed American psychiatrists’ attitudes on hallucinogens^29^, while Wildberger et al. assessed the opinions of college students^30^. Corrigan et al. surveyed patients’ opinions on psilocybin therapy^31^. A study by Davis et al. assessed American psychologists’ opinions on psychedelics^32^, while Hearn et al. surveyed counselors in the United States^33^. All studies have so far used unvalidated self-developed questionnaires and presented their results as a cross-sectional overview of responses for each item. No validated instrument for quantifying attitudes on psychedelics currently exists.

Therefore, the aim of this study was to develop and test the psychometric properties of a questionnaire to assess attitudes on psychedelics, the Attitudes on Psychedelics Questionnaire (APQ). We also aimed to explore the relationship between APQ scores and basic knowledge on psychedelics.

## Results

### Demographic information

The final analysis included n = 1153 participants (response rate 69.2%). From n = 1667 participants that started filling out the survey, n = 514 participants were excluded according to pre-defined exclusion criteria (see Supplementary Figure D.1 in Appendix D). Female participants were in the majority (n = 716, 62.1%), and most participants had completed either a graduate/university (n = 429, 37.2%) or high school degree (n = 398, 34.5%) (see Supplementary Table D.1 in Appendix D). Median (Md) participant age in years was Md = 31 (IQR = 23–40). Health care workers constituted 15.5% of participants (n = 179), and a majority of them were physicians (n = 108, 60.3%) (see Supplementary Tables D.1-D.2 in Appendix D).

### Attitudes on psychedelics (APQ) questionnaire

#### Pilot survey

There were no items with a mean score < 2.0 or > 4.0. We kept a similar number of positively and negatively worded items for each sub-scale, as well as items that clearly belonged to sub-scales by their meaning. We took into account that a minimum of 2 items for each of the affective, behavioural and cognitive categories within the tripartite model of attitudes was included. We also examined inter-item correlations and item-total correlations, the reliability of the total scale and within specific sub-scales, as well as the reliability score if an item is dropped, where a significant increase was defined as that of at least 0.05. This was performed as part of an iterative process that followed the removal of individual items, with continuous modification and re-assessment of both these variables and face validity of items and scale structure until a satisfactory proposed model of the questionnaire was constructed. One hypothesised sub-scale (*Prejudices on Psychedelics*, 4 items) was removed due to low reliability. The result of the pilot survey was a hypothesized version of the APQ, consisting of 20 items (2 affective, 4 behavioural, 14 cognitive) within 4 sub-scales of 5 items each (see Table 1). The items in Croatian are provided in Suppl. Table F.1 in Appendix F.

**Table 1:** The hypothesized model of the APQ in English.

	Item nos	Item text
**Sub-scale**		
Legal Use of Psychedelics	1	Legalizing psychedelics would benefit public health.
	2	Those who want to legalize psychedelics have a hidden agenda behind their actions. (R)
	3	The use of psychedelics for justified medical reasons should be legal.
	4	Administering psychedelics to psychiatric patients is safe as long as the treatment conditions are carefully controlled.
	5	Administering psychedelics to patients will eventually lead to bad outcomes. (R)
Effects of Psychedelics	6	Psychedelic use is linked to creativity.
	7	If more people used psychedelics, the world would be a better place.
	8	Recreational use of psychedelics has no practical benefit. (R)
	9	I am afraid of the effects of psychedelics on physical health. (R)
	10	Psychedelics can provide valuable spiritual experiences.
Risk Assessment of Psychedelics	11	Using psychedelics is safe.
	12	The use of psychedelics can damage the nervous system. (R)
	13	Psychedelics are less dangerous than other illegal drugs.
	14	A wider use of psychedelics would cause an increase in mental problems. (R)
	15	Administering psychedelics to patients is not problematic as long as it is performed by a professional.
Openness to Psychedelics	16	I am optimistic about psychedelic research.
	17	I would not agree to use psychedelics for mental health purposes. (R)
	18	If psychedelic-assisted psychotherapy enters into regular practice, I would be interested in learning more about it.
	19	I would be interested in learning about other people’s experiences with psychedelics.
	20	I don’t think that learning about psychedelics is worth my time. (R)

Negatively worded items that are reversely coded are marked by (R).

#### Construct validity and comparison of models

We conducted a confirmatory factor analysis (CFA) on the following structural models:

1. The hypothesized 4-factor model structure;
2. A hierarchical 4-factor model, i.e. a version of the 4-factor model that included a second-order factor accounting for covariance between first-order factors;
3. A 3-factor model, constructed by observing the highest factor covariance (0.935) in the hypothesized model (*Legal Use of Psychedelics* and *Risk Assessment of Psychedelics* were constrained into a single factor);
4. A 2-factor model, constructed by again observing the highest factor covariance in the 3-factor model (*Legal Use of Psychedelics*, *Risk Assessment of Psychedelics*, and *Effects of Psychedelics* were constrained into a single factor).

Nested models 2–4 were compared to the hypothesized 4-factor model (see Table 2).

**Table 2:** Model fit indices for all assessed nested structural models of the APQ.

Model	RMSEA (95% CI)	SRMR	CFI	TLI	χ^2^ (df)	Δχ^2^/Δdf^a^	ΔRMSEA^b^
**4-Factor model**	**0.042 (0.038–0.046)**	**0.054**	**0.992**	**0.991**	**496.16 (164)**	**–**	**–**
Hierarchical 4-factor model	0.043 (0.039–0.047)	0.055	0.991	0.990	518.95 (166)	+ 22.79/+ 2	+ 0.001
3-Factor model	0.044 (0.039–0.048)	0.056	0.991	0.990	531.52 (167)	+ 35.36/+ 3	+ 0.001
2-Factor model	0.046 (0.042)	0.058	0.990	0.989	575.17 (169)	+ 79.01/+ 5	+ 0.002

The model with the best fit is shown in bold. N = 1153.

RMSEA, root mean square error of approximation; CI, confidence interval; SRMR, standardized root mean squared residual; CFI, Comparative Fit Index; TFI, Tucker-Lewis Fit Index; χ^2^, Chi-square; df, degrees of freedom.

^a^Likelihood ratio test, shown as a change in χ^2^/df values relative to the parent model i.e. the hypothesized 4-factor model (top row).

^b^Change in RMSEA value relative to the parent model i.e. the hypothesized 4-factor model (top row).

All structural models showed acceptable fit. However, the hypothesized 4-factor model showed lowest Root Mean Square Error of Approximation (RMSEA) values and a lower likelihood ratio test (χ^2^/df) result relative to other models, which was considered more favourable (see Suppl. Appendix C).

#### Reliability analysis

The 20 items from the APQ showed excellent total scale reliability (McDonald’s omega (ω) = 0.949, 95% CI = 0.944–0.953), as did all sub-scales in the hypothesized 4-factor model: *Legal Use of Psychedelics* (ω = 0.842, 95% CI = 0.828–0.856), *Effects of Psychedelics* (ω = 0.881, 95% CI = 0.870–0.892), *Risk Assessment of Psychedelics* (ω = 0.841, 95% CI = 0.826–0.855), and *Openness to Psychedelics* (ω = 0.843, 95% CI = 0.829–0.858) (see Supplementary Table E.1 in Appendix E).

#### Convergent validity

The hypothesized 4-factor model had good convergent validity, as its total score was shown to highly correlate with the total score on the Barnett et al. questionnaire (r = 0.885, P < 0.001). All of the sub-scales on the APQ did as well: *Legal Use of Psychedelics* (r = 0.859, P < 0.001), *Effects of Psychedelics* (r = 0.792, P < 0.001), *Risk Assessment of Psychedelics* (r = 0.822, P < 0.001), and *Openness to Psychedelics* (r = 0.736, P < 0.001).

#### Best model

**Figure 1:**
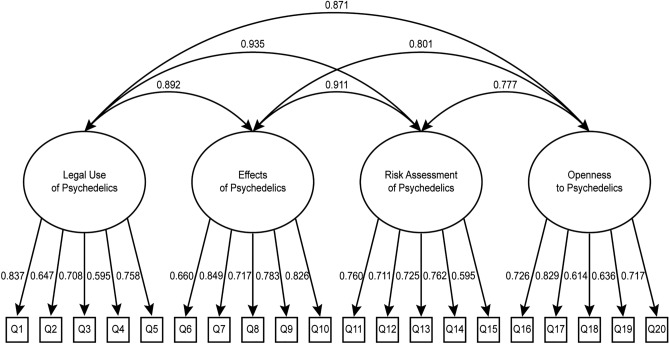
Structural representation of the final model of the APQ, with factor covariances and standardized item loading estimates for each factor.

Our analysis showed that the best model of the APQ is the initially hypothesized 20-item questionnaire with 4 sub-scales and a theoretical total score range of 20–100, and a theoretical score range of 5–25 for each sub-scale (see Table 1). Even though we did not choose the hierarchical 4-factor model, we think that a calculation of the scale total using the sum of scores on all sub-scales is justified due to high observed factor covariances (see Fig. 1). All unstandardized and standardized factor loading estimates are reported in the Supplementary Table E.5.

### Attitudes on psychedelics scores

The median total score on the APQ was Md = 65.0 (IQR = 56.0–77.0, 95% CI = 64.0–66.0). Median scores on sub-scales were as follows: *Legal use of Psychedelics* Md = 17.0 (IQR = 15.0–20.0, 95% CI = 17.0–18.0), *Effects of Psychedelics* Md = 15.0 (IQR = 11.0–19.0, 95% CI = 14.0–15.0), *Risk Assessment of Psychedelics* Md = 15.0 (IQR = 12.0–17.0, 95% CI = 14.0–15.0), and *Openness to Psychedelics* Md = 19.0 (IQR = 16.0–22.0, 95% CI = 19.0–20.0).

The median score on the Barnett et al. questionnaire (scale range 7–35) was Md = 23.0 (IQR = 16.0–30.0, 95% CI = 23.0–24.0). The item and factor structure of the modified Barnett et al. questionnaire showed satisfactory psychometric properties (see Supplementary Tables E.2-E.4 in Appendix E).

### Basic knowledge on psychedelics scores

The median score on the knowledge on psychedelics test was Md = 63.6 (IQR = 50.0–81.8, 95% CI = 64.9–68.2). The three most commonly correctly recognized psychedelics were LSD (n = 1038, 90.0%), MDMA (n = 866, 75.1%), and psilocybin (n = 829, 71.9%). Three substances most commonly mistaken for psychedelics were opium (n = 690, 59.8%), methamphetamine (n = 665, 57.7%), and heroin (n = 543, 47.1%). Responses for each substance are shown in Supplementary Table D.4 in Appendix D.

### Additional analyses

Participants excluded from the study did not differ from those included by gender distribution or age (see Supplementary Table D.3 in Appendix D). Post-hoc Chi-square test analysis showed no significant difference in educational characteristics of the groups (see Supplementary Table D.3 in Appendix D). From n = 447 participants that were excluded because they didn’t complete the whole survey, 43.4% (n = 194) gave up during the knowledge on psychedelics test. Feedback from participants also indicated that some dropped out from the survey at the knowledge test because it became clear to them that they cannot properly respond due to inadequate knowledge on psychedelics (see Suppl. Appendix B).

We observed a positive correlation between total scores on knowledge and attitudes on psychedelics (r = 0.494, P < 0.001). Male gender (standardized regression coefficient (β) = − 0.171, P < 0.001), younger age (β = − 0.218, P < 0.001), and lower educational status (β = − 0.124, P < 0.001) showed an association with higher APQ scores, but together explained only 12.6% of score variance. HCW status was not associated with APQ scores (β = − 0.049, P = 0.091), but was with *Legal use of Psychedelics* and *Effects of Psychedelics* sub-scale totals (see Supplementary Table D.5 in Appendix D).

Non-HCW participants (n = 974) and HCWs (n = 179) did not significantly differ in knowledge on psychedelics scores (P = 0.711), but non-HCWs had slightly more positive attitudes on psychedelics scores than HCWs (P < 0.001) (see Supplementary Table D.6 in Appendix D). Median scores of responses to items that were only for HCWs show a trend of being neutral towards referring patients to psychiatrists that use psychedelics or support their legalization. There was low concern with prescribing or recommending psychedelics to patient if their efficacy and safety is proven, as well as a general interest towards witnessing sessions of psychedelic-assisted psychotherapy (see Supplementary Table D.7 in Appendix D).

### Deviations from the protocol

We renamed the correlation between the Barnett et al. questionnaire and the APQ from criterion to convergent validity, as it was more appropriate. We chose not to set any cut-offs for APQ scores and did therefore not define any of the findings as positive or negative attitudes on psychedelics.

## Discussion

Our study describes the development and initial psychometric properties of a new scale, the APQ, which measures attitudes on psychedelics. Confirmatory factor analysis has demonstrated our new instrument’s construct validity and confirmed a factor structure of four sub-scales: *Legal Use of Psychedelics*, *Effects of Psychedelics, Risk Assessment of Psychedelics*, and *Openness to Psychedelics*, all of which demonstrated a high internal consistency, as did the overall APQ scale. Convergent validity was supported by a strong correlation with the score on the modified Barnett et al. questionnaire for assessment of attitudes on psychedelics, which also showed good psychometric properties. LSD, MDMA, and psilocybin were the most widely recognized psychedelics in the basic knowledge test, whereas opium, methamphetamine, and heroin were most often mistaken to be psychedelics. Knowledge on psychedelics scores positively correlated with APQ scores. Younger age, male gender, and lower educational status were associated with more positive attitudes on psychedelics. HCW status was only associated with more negative attitudes on the legal status of psychedelics and the perception of their effects, but general response trends in this population showed openness and curiosity in regards to psychedelic-assisted psychotherapy. There was no difference in basic knowledge on psychedelics between HCWs and the lay population.

Our findings were generally consistent with the existing literature on attitudes on psychedelics. An association of younger age and male gender with more positive attitudes on psychedelics that we found was also previously observed by Barnett et al.^29^ and Hearn et al.^33^. Likewise, Reynolds et al. conducted a qualitative study involving HCWs working with terminally ill patients and observed that an interviewee’s knowledge influences the way they talk about and perceive psychedelics^34^, thus the correlation of knowledge and attitudes on psychedelics that we observed confirms and quantifies their field observations. A strong correlation of APQ scores with the questionnaire on attitudes on psychedelics previously developed by Barnett et al. strengthens the validity of our findings, especially since the Barnett et al. items also showed good psychometric properties in this study^29^. The added value of the APQ is in its wider and more detailed scope and that it allows the use of each of its specific sub-scales individually. Although cognitive items make up the majority of the APQ, it also includes items that address the behavioural and affective components of attitudes on psychedelics.

Davis et al. showed that even mental health professionals have limited knowledge on psychedelics^32^, which was also evident in our study, where HCWs had no better knowledge than the lay population. It is also not surprising that many of our participants had misconceptions about which substances are psychedelics. A survey of college students’ attitudes on hallucinogens showed that a majority of them thought that hallucinogens cause addiction^30^, even though their dependency potential is generally known to be low^35–38^. Poor general knowledge on psychedelics is understandable, as psychedelic research has only recently experienced revival and the information on these topics is slowly reaching the mainstream^3,39^. A high percentage of our participants gave up at the knowledge test and this was also reflected in their feedback where those who dropped out plainly stated that did not have enough knowledge to answer the survey. This indicates that the APQ cannot be administered to participants who have almost no knowledge at all on psychedelics. The distribution of knowledge scores in our study on a scale from 0 to 100 indicate that half of participants had average to above-average knowledge of psychedelics. Nevertheless, a significant number of our participants thought that drugs such as heroin belong to the psychedelic group, indicating a poor understanding of psychedelics’ effects and the classification of illicit substances in general. However, by showing our participants the correct answers to the test, we made sure that their answers are representative and indeed reflect attitudes on psychedelics.

The main limitation of our study was the risk of selection bias, as is common with survey studies. We attempted to avoid this by sampling a wide demographic of participants, sending reminders to invitees, emphasizing that all opinions are equally valuable for our survey, and providing the survey in the participants’ native language. There is, however, a disclaimer needed regarding the generalizability of our survey results, as Croatia is ethnically and culturally a very homogeneous country. We encourage future studies to conduct a validation and analysis of APQ scores in ethnically, geographically and culturally diverse settings, as our results are valid only within the Croatian context. Additionally, the snowballing sampling method did not allow us to determine the true response rate, as we cannot be sure how many invited participants did not access our survey or what their demographic profile is. However, for those who did access the survey, an analysis of attrition bias showed that participants that gave up at some point in the survey were not different in terms of demographic data from those who did not complete the survey. Because of this, we were careful not to attempt to describe or set any cut-off values for our instrument, as we cannot claim complete representativeness of our target sample. The snowballing sampling design was still very useful, as it allowed us to reach a large sample size, which decreased the risk of selection bias, increased the external validity of our findings, and can be considered excellent for an initial validation study^40^. We did not assess self-reported knowledge on psychedelics as was done in previous studies on attitudes on psychedelics^30,32^, but used an objective quantitative method of knowledge testing that is not open to over- or underestimating one’s knowledge due to universal cognitive biases such as the Dunning-Kruger effect^41^. Additionally, we cannot claim that our initial item pool for the APQ was fully comprehensive, as there may be other unknown aspects not captured through our instrument. However, we used the expert panel assessment and a clear theoretical framework during item generation and selection to ensure that a wide range of relevant aspects is covered.

There are several ways how the APQ can be applied further. The study by Davis et al. found that psychologists in the US are interested in psychedelic as mental health treatments, but report only partial knowledge of their properties and characteristics, so the authors highlighted the importance of educating this population^32^. Our findings of the association between knowledge and attitudes on psychedelics strongly indicate that assessing educational interventions is a logical next step, where the APQ has the potential to provide useful before-and-after and between-group comparisons. It can also give information on which groups would benefit most from educational interventions and which aspects their curricula should focus on. Likewise, it is known that participants in trials involving psychedelics are subject to an expectancy bias and various extra-pharmacological factors which affect the intensity of the psychedelic experience^42,43^. Assessing baseline attitudes on psychedelics in participants enrolled into psychedelic-assisted psychotherapy trials could provide insight if treatment response or intensity of the psychedelic experience is associated with their pre-existing attitudes and beliefs. As it is difficult to obtain a sample truly representative of the general population, future studies should define cut-offs for APQ scores by applying the questionnaire in specific populations, such as psychologists or psychiatrists. There is currently a growing body of literature on attitudes on cannabis exploring harm perception and attitudes of various population groups as a proxy measure of cannabis use^44–46^. Our instrument could be similarly used to provide context surrounding the introduction of psychedelics as a form of therapy. APQ scores could serve as a proxy measure of previous experience with or willingness to use or, in the case of mental health professionals, to prescribe psychedelics. As with cannabis, trends in attitudes on psychedelics could also foreshadow future policy and legislation changes^45^. In the case of mental health experts such as psychiatrists, an association between different sub-scales of the APQ and willingness to use or prescribe psychedelics could be explored to understand factors influencing these phenomena. We did not ask our participants about previous recreational use of psychedelics due to concerns about selection bias or socially desirable responses which could skew the results in our study, whose primary aim was validation. Demographic variables in this study only explained a small amount of APQ score variance, so psychedelic use may be an important unexplored predictor of attitudes on psychedelics. Future studies should consider exploring whether self-reported recreational psychedelic use, as well as other demographic factors, are associated with APQ scores. For example, as psychedelic use can alter core metaphysical beliefs, it would be useful to see if spirituality or religiosity are also associated with attitudes on psychedelics^47^. We encourage authors in other ethnically diverse settings to explore ethnicity as an important demographic factor, as well. There is a potential for very different cultural perceptions of psychedelics, especially considering the long history of indigenous use of psychedelics^48^. Additionally, participants’ sexual orientation may be a relevant factor influencing attitudes as measured by the APQ and should be considered in future analyses. To sum up, there are multiple voices and perspectives about psychedelics in the public sphere, ranging from the example of author and journalist Michael Pollan with the book and TV series “How to Change Your Mind” as a positive representation of psychedelics^49^, to the concerning ethical issues related to psychedelic therapy brought up in “Cover Story: Power Trip”, an investigative podcast series by the New York Magazine^50^. Within this polarized landscape, an instrument such as the APQ could help provide a wider perspective that goes beyond individual cases.

This study demonstrated the reliability and validity of the APQ in assessing attitudes on psychedelics, something that will continue to be widely explored and assessed due to the ongoing revival of psychedelic research. We encourage the replication and exploration of our new instrument in different settings and populations. Although the initial validation was in Croatian, we provide herein a valuable tool for future widespread use and thus encourage further validation and use of the APQ in English.

## Methods

### Study design

This was an observational, cross-sectional study. The study was pre-registered at the Open Science Framework (available online at https://osf.io/mj96r).

### Development of the APQ

#### Item creation

An item pool of 122 original items reflecting attitudes on psychedelics was generated in English. The authors first held a preliminary discussion on relevant aspects to be represented in the item pool. The most important aspects were identified as: opinions on the effects of psychedelics, legalization, medical uses of psychedelics, behavioural openness (willingness to use psychedelics or learn more about them), and prejudice about users of psychedelics. We used the tripartite model of attitudes to guide item creation from a theoretical standpoint. The model states that an attitude is composed of three aspects: affective, behavioural, and cognitive^51^. Thus, we made sure that we represented all these aspects within the item pool. For example, the item “I am afraid of the effects of psychedelics on physical health” carries an affective component, “I would not agree to use psychedelics for mental health purposes” refers to behavioural intent regarding psychedelics, while “The use of psychedelics can damage the nervous system” reflects a cognitive belief. The item pool was then sent to four experts for review of face validity: a research methodology expert, a psychiatrist, a language expert and an epidemiologist. The experts were selected because they had background training of at least a master’s degree and expertise in the aforementioned disciplines, which we considered to be relevant to questionnaire development and the subject of psychedelics. We asked the experts to evaluate the items with respect to clear wording, ambiguity, understandability, proper terms and grammar, and relevance. They could add comments on any of the items and suggest new items or topics if needed. Following their input, 39 items were excluded and no new items were generated. We were left with a pool of 83 items (8 affective, 14 behavioural, 54 cognitive), with responses given on a 5-point Likert-type scale ranging from 1—“Completely disagree” to 5—“Completely agree”. We chose to present items in Croatian to participants in the pilot and validation surveys, since using items in English would introduce bias by selecting for participants who are proficient in English (e.g. highly educated, younger participants). The English items were translated by one author (MFŽ) into Croatian, and a language expert uninvolved in the study back-translated the items into English to ensure translation accuracy.

#### Pilot survey

We then conducted a pilot survey on N = 116 students of the Faculty of Humanities and Social Sciences in Split (demographic data shown in Supplementary Table A.3 in Appendix A). The survey was in Croatian and participants filled it out online.

#### Validation survey

We recruited a sample of the Croatian general population using a convenience snowballing sampling method. It was in Croatian and participants filled it out online via the SurveyMonkey platform (SurveyMonkey Inc., San Mateo, CA, USA). Data were collected between July 20, 2021 and November 1, 2021. An invite to the survey was disseminated by nine different groups and associations to its members (full information shown in Suppl. Appendix B). Additionally, all authors disseminated the survey individually, using social media and personal contacts.

The inclusion criteria were that the participant is over 18 years of age. We included both lay persons and health care workers (HCWs). Participants that did not complete all parts of the survey were excluded, thereby avoiding any issues with missing data. We also excluded all participants with a survey completion time over 45 min to decrease the chance of participants reading about psychedelics during the survey. We considered this a reasonable maximum time for survey completion, allowing participants roughly a minute per each question within the questionnaires and the knowledge test. All outliers in attitude scores were included in the analysis, as we believed they may reflect genuine extremely negative or positive attitudes of participants. Only unique visitors were allowed to fill out the survey.

The survey was anonymous and consisted of three parts: demographic information, basic knowledge on psychedelics survey, and the attitudes on psychedelics survey (with items from the Barnett et al. questionnaire and our hypothesized 20-item APQ). Items within each section were presented to participants in a randomized order. We collected the following demographic information: participants’ age, gender, highest attained level of education, whether the participant is a HCW, and (for HCWs) type of profession. As Croatia is a highly homogenous country in terms of ethnicity (90% Croats, 4% Serbs, and all other ethnicities < 1%), we did not ask participants for their ethnicity. Likewise, the question of sexual orientation was not considered as a potential variable, as the report from the University of California, Berkeley listed Croatia among the top ten percent of worldwide countries in terms of social inclusivity for 2020 (13th out of 134 analysed countries)^52^. HCW participants were presented with an additional 5 questions (not part of the APQ) on their attitudes on psychedelics that relate to their healthcare practice (see Supplementary Table A.4 in Appendix A).

### Modification of the Barnett et al. questionnaire

We included 7 items from a prior study on attitudes on psychedelics by Barnett et al.^29^ for the assessment of convergent validity. Their items were not validated as a scale, but they were most similar to the purpose of the APQ. Since Barnett et al. used the term “hallucinogens” in their study, we kept item wording the same, but changed “hallucinogens” into “psychedelics” (see Supplementary Table A.1 in Appendix A).

### Basic knowledge on psychedelics test

We created a brief test on the basic knowledge of psychedelic substances to test how accurately participants were able to correctly classify substances as psychedelics, and to subsequently ensure that their answers in the attitudes on psychedelics questionnaires actually refer to psychedelics. There is a lot of varied use of the term “psychedelic” and some contention on which substances to include in this group and why. In our survey, psychedelics were thus defined under a broader definition that included MDMA and ibogaine, in addition to classic psychedelics^1^. Our rationale was primarily based on the similarity in chemical structure between these two substances and classical psychedelics^17,22^. We did not define ketamine as a psychedelic due to its widely used classification as a dissociative, differences in its chemical structure, as well as its primarily glutamatergic receptor effects^19^. The full basic knowledge on psychedelics test and a detailed description of its methodology and development are provided in Supplementary Table A.2 in Appendix A.

### Ethical considerations

The ethics approval for the study was obtained from the Ethics Committee of the USSM (document No. 2181-198-03-04-21-0077). The study was conducted in accordance with all regulations of the USSM Ethics Committee and the Declaration of Helsinki. The survey was anonymous and no IP addresses were collected. Participants provided informed consent to both the pilot and validation survey by checking a box to confirm their participation after being provided basic information on the study. They were free to stop filling out the survey at any time and received no incentives to participate.

### Statistical analysis

All statistical tests were performed using JASP v. 0.14 (JASP Team, 2020, Amsterdam, Netherlands), SPSS Statistics v.22.0 (IBM, Armonk, NY, 2013), R software v.4.1.1. (R Foundation for Statistical Computing, 2021, Vienna, Austria) and MedCalc v. 19.5.3 (MedCalc Software Ltd, 2020, Ostend, Belgium) software packages. Normality of data distribution was assessed using the Shapiro–Wilk test. The cut-off for P-value significance was < 0.05 in all tests.

#### Demographic information

All demographic information was shown as frequencies and percentages (N, %) except for age, which was shown using median (Md) and interquartile range (IQR). The response rate was calculated as the number of participants with a fully completed survey divided by the total number of participants who accessed the survey and either completed it or stopped filling it out before completion.

#### Attitudes on psychedelics (APQ) questionnaire

##### Pilot survey

Content examination of 83 items was performed using principal component analysis to group items into sub-scales that reflect different aspects of attitudes to psychedelics. We examined inter-item correlations, item-total correlations, and sub-scale and total reliability. We calculated a mean score for each item to see if any had a deviation of response distribution to either extremely negative or positive answers.

##### Construct validity and comparison of models

A CFA was performed for different structural models of the APQ to assess construct validity. This was done using the diagonally weighted least squares (DWLS) estimation method with a polychoric correlation matrix, as recommended by Li et al.^53^. Results were assessed using the following model fit indices and corresponding cut-off values for acceptable model fit^54^: RMSEA ≤ 0.06, Standardized Root Mean Squared Residual (SRMR) ≤ 0.08, Comparative Fit Index (CFI) ≥ 0.95, and the Tucker-Lewis Fit Index (TFI) ≥ 0.95. RMSEA was expressed using a 95% confidence interval (CI). We used the likelihood ratio test (Δχ^2^/Δdf) as a method of comparing model fit^40^. A description and rationale for all model evaluation methods is available in Suppl. Appendix C.

##### Reliability analysis

Reliability analysis was performed using McDonald’s ω and 95% CI for the model with the best fit. We used ω instead of Cronbach’s α since the assumption of tau-equivalence was violated in our case^55^.

##### Convergent validity

To assess convergent validity, we estimated the correlation (expressed as Pearson’s r and a P-value) between the APQ model with the best fit (and its sub-scales) with the score on our modified version of the Barnett et al. questionnaire^29^. Its psychometric properties were evaluated by an EFA with oblimin rotation, reliability analysis, and CFA to assess model fit.

#### Attitudes on psychedelics scores

Scores on the APQ and each of its sub-scales, as well as the modified Barnett et al. questionnaire were shown using Md, IQR and 95% CI. All scale and sub-scale totals were calculated as the sum of all items with no weights applied.

#### Basic knowledge on psychedelics scores

Scores from the basic knowledge test were shown using Md, IQR and 95% CI. The number of correct or incorrect responses for each substance was shown using frequencies and percentages.

#### Additional analyses

To address potential attrition bias, we compared demographic information between included and excluded participants using the Mann–Whitney (for age) and Chi-square tests (for gender and education level). We used Pearson's r to estimate the correlation between knowledge on psychedelics scores and scores on the APQ. Linear regression modelling was performed, with gender, age, education level and HCW status (yes/no) as covariates and APQ scores and each of its sub-scales as dependent variables, respectively. Results were expressed as standardized regression coefficients (β), P-values, and coefficients of determination (R^2^).

We conducted a subgroup analysis by using the Mann–Whitney test to compare knowledge and attitude scores between HCWs and non-HCW participants. Items for HCWs had responses for each item presented using Md, IQR, and 95% CI.

#### Sample size calculation

We calculated a minimum sample size of 385 participants by using a sample size calculator set to an unlimited population, a 95% confidence level, 5% margin of error, and a population proportion of 50%^56^.

### Protocol registration

The study was pre-registered at the Open Science Framework (available online at https://osf.io/mj96r).

## Supplementary Information

Supplementary Information 1.

Supplementary Information 2.

Supplementary Information 3.

Supplementary Information 4.

Supplementary Information 5.

Supplementary Information 6.

## Data Availability

The dataset used in this study is publicly accessible at the Open Science Framework, https://doi.org/10.17605/OSF.IO/TVRHQ.
