# Supplementary material for: Validation of a new instrument for assessing attitudes on psychedelics in the general population
Source: Sci Rep. 2022 Oct 29;12:18225. doi: 10.1038/s41598-022-23056-5 (PMC9617880; doi:10.1038/s41598-022-23056-5)
Supplement: Supplementary file 1 — Supplementary Information 1. [file 41598_2022_23056_MOESM1_ESM.docx]

**Appendix A: Modified Barnett et al. items, basic knowledge on psychedelics survey, information on pilot survey demographics, survey items for health care workers**

**Supplementary Table A.1**. Modified items by Barnett et al. that were included in our survey. Negatively worded items that are reversely coded are marked by (R).

| **Item no.** | **Item text** |
| --- | --- |
| 1 | The use of psychedelics increases the risk for subsequent psychiatric disorders. **(R)** |
| 2 | The use of psychedelics increases the risk for long-term cognitive impairment. **(R)** |
| 3 | The use of psychedelics should be illegal for recreational purposes. **(R)** |
| 4 | The use of psychedelics is unsafe even under medical supervision. **(R)** |
| 5 | The use of psychedelics shows promise in treating psychiatric disorders. |
| 6 | The use of psychedelics may improve outcomes when used during psychotherapy. |
| 7 | The use of psychedelics deserves future research for the treatment of psychiatric disorders. |

**Basic knowledge on psychedelics test**

We developed a list of 22 substances (psychedelics, stimulants and other drugs of abuse, various psychotropic medications), of which 7 were psychedelics. Participants were presented with the substances in a randomized order and asked to mark “Yes” or “No” next to each substance. “Yes” meant that they believe that the substance belongs to the group of psychedelics. After submitting their answers by proceeding in the survey, they were unable to modify them further, and were then presented with the correct answers on the following page, as well as additional information (pharmacological class, mechanism of action) for each non-psychedelic substance. In this sense, the knowledge test was also a brief educational intervention. We decided not to include an “I don’t know” response option on the test as we believed that many participants would avoid answering if presented with this option. The yes/no forced choice question design allowed us to more clearly evaluate any misconceptions participants may have about psychedelics (such as confusing psychedelics with another class of drugs that have different properties, e.g. heroin is a psychedelic drug). We could not make sure that participants would not use the Internet to read about psychedelics during the survey, but all participants who filled out the survey for a long time (>45 minutes) were excluded as a precaution.

The full basic knowledge tests appeared as follows:

**Supplementary Table A.2**. Basic knowledge on psychedelics test used in this study. Substances marked in bold are psychedelics. Substances were shown to each participant in a randomized order.

| Substance | Response options – whether the substance is a psychedelic or not | |
| --- | --- | --- |
| Cocaine | Yes | No |
| **LSD** | Yes | No |
| **Psilocybin** | Yes | No |
| Imipramine | Yes | No |
| Heroin | Yes | No |
| **Ibogaine** | Yes | No |
| Phenobarbital | Yes | No |
| Methamphetamine | Yes | No |
| **MDMA (ecstasy)** | Yes | No |
| **DMT** | Yes | No |
| Digoxin | Yes | No |
| **Mescaline** | Yes | No |
| Modafinil | Yes | No |
| Ketamine | Yes | No |
| Haloperidol | Yes | No |
| Dextroamphetamine | Yes | No |
| Gamma-hydroxybutyrate (GHB) | Yes | No |
| **Peyote** | Yes | No |
| Rohypnol | Yes | No |
| Oxycodone | Yes | No |
| Opium | Yes | No |
| Mexazolam | Yes | No |

**Correct answers**

After participants marked their answers on the survey, they moved on to the next page in SurveyMonkey and were presented with the following text:

“From the substances presented in the previous question, the following belong to the group of psychedelic substances (psychedelics): Lysergic acid diethylamide (LSD), psilocybin, ibogaine, MDMA (ecstasy), DMT, mescaline, peyote.

Other substances from the list do not belong to psychedelics: cocaine (stimulant), imipramine (tricyclic antidepressant), heroin (opioid), phenobarbital (barbiturate sedative), methamphetamine (stimulant), digoxin (cardiac glycoside), modafinil (eugeroic i.e. wakefulness-promoting drug), ketamine (anaesthetic, NMDA receptor antagonist in the brain), haloperidol (antipsychotic), dextroamphetamine (stimulant), gamma-hydroxybutyrate (GHB) (central nervous system depressant), rohypnol (benzodiazepine sedative), oxycodone (opioid), opium (natural substance used to produce opioid drugs), mexazolam (benzodiazepine sedative).

**Scoring**

The total knowledge on psychedelics score was determined by the number of incorrect answers (a non-psychedelic incorrectly identified as a psychedelic) subtracted from the number of correct answers (correctly identified psychedelic). We chose this calculation method because it prevented a participant from getting the highest score by marking all substances as psychedelics. The theoretical scale range was from -15 (all answers incorrect) to 7 (all answers correct). To ease the interpretation of scoring on the knowledge on psychedelics survey, we converted scores from a scale from -15 to 7 to a scale of 0-100 using a method which was previously used for the CIVIQ questionnaire (1).

We used the following formula:

*(S-m)/(M-m)*100*

where S – the total score/result, m – minimum theoretical value, M – maximal theoretical value.

In our case (scale range -15 to 7), the formula applied to each participant’s score (S) in knowledge on psychedelics was:

*(S-(-15)/(7-(-15))*100=(S+15)/(7+15)*100*

**Supplementary Table A.3**. Descriptive analysis of demographic information of study participants in the pilot survey. N=116.

| **Variable** | **n (%)** |
| --- | --- |
| **Gender** | |
| Male | 21 (17.4) |
| Female | 95 (77.7) |
| Undisclosed | 1 (0.8) |
| **Year of study** | |
| 1^st^ | 2 (1.7) |
| 2^nd^ | 16 (13.2) |
| 3^rd^ | 7 (5.8) |
| 4^th^ | 58 (47.9) |
| 5th | 20 (16.5) |
| Other/does not apply to me | 13 (10.7) |

**Supplementary Table A.4**. Additional items for the health care workers’ subgroup of survey participants. Negatively worded items that are reversely coded are marked by (R).

| **Item no.** | **Item text** |
| --- | --- |
| 1 | I would be worried if my institution took part in research with psychedelics. **(R)** |
| 2 | I would feel uncomfortable recommending psychedelic treatment to a patient, even if there is evidence for its effectiveness and safety. **(R)** |
| 3 | As a health professional, I would be interested in witnessing a psychedelic session with a patient. |
| 4 | If I knew that a psychiatrist used psychedelics, I would be less likely to refer my patient to them. **(R)** |
| 5 | If I knew a psychiatrist who supported the legalization of psychedelics, I would be less likely to refer my patient to them. **(R)** |

Note:

**References**

1. CIVIQ Users’ Guide. Scoring and missing data. [accessed November 17, 2021]. Available from: https://www.civiq-20.com/scoring-missing-data/
