# Supplementary material for: Validation of a new instrument for assessing attitudes on psychedelics in the general population
Source: Sci Rep. 2022 Oct 29;12:18225. doi: 10.1038/s41598-022-23056-5 (PMC9617880; doi:10.1038/s41598-022-23056-5)
Supplement: Supplementary file 2 — Supplementary Information 2. [file 41598_2022_23056_MOESM2_ESM.docx]

**Appendix B: Sampling methods, survey response trends and participant feedback**

**Sampling methods and survey response trends**

Our survey was disseminated within the following organizations or social media groups between July and October 2021:

- CroMSIC Split, a **medical student association** (disseminated on their Facebook page to around 1400 followers on July 28);
- SplitMisli, a **youth and student association** (disseminated on their Facebook page to around 1300 followers on October 14);
- Udruga ATMA, an **independent lifestyle news portal and association** (disseminated on their Facebook page to around 207.000 followers on October 14);
- Udruga MoSt, a **non-governmental, non-profit organization with aid programs for the homeless and impoverished** (disseminated to their members via mailing list on October 14, number unknown);
- The Croatian Association for the Promotion of Patients' Rights, a **non-governmental, non-profit patients’ rights organization** (disseminated on their Facebook page to around 10.300 followers on October 19);
- Psihodelični Vrt, a **closed Facebook group for discussion on psychedelics** (disseminated within the Facebook group to around 1700 members on October 27);
- Psihodelično društvo Balkana, an **open Facebook group for discussion on psychedelics** (disseminated within the Facebook group to around 650 members on October 27);
- Soba, a **closed Facebook group for underground clubbing events** (disseminated within the Facebook group to around 1700 members on October 27);
- Nepopularna Psihologija, **a psychology blog and website** (disseminated on their Facebook page to around 10.200 followers on October 28).

As mentioned above, dissemination in groups related to psychedelics happened on October 27, 2021. We see a rise in survey responses around this date after another previous peak (see **Supplementary Figure B.1**). However, as our sampling method is snowballing, we are unable to pinpoint if this is only due to members from these groups responding more to the survey (or also from the psychology blog and website that advertised the survey a day later). An upward trend of responses is visible around this time period, but all images of response trends in this document are informative only.

**Supplementary Figure B.1**. Frequency of survey responses by day for the time period from October 3 to November 1, 2021.


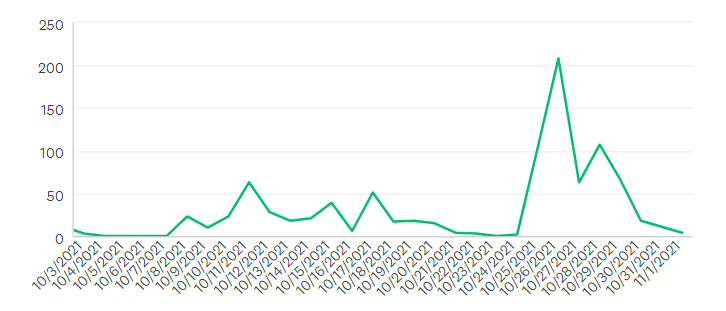


To contrast the response trends from October, we show the second largest peak in responses per day at the beginning of data collection, in July (see **Supplementary Figure B.2**).

**Supplementary Figure B.2**. Frequency of survey responses by day for the time period from October 3 to November 1, 2021.


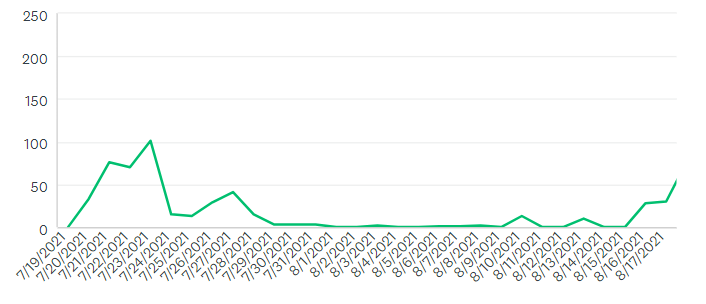


**Participant feedback**

When we sent out the survey, we received feedback from some participants directly, or the feedback was provided to the person disseminating the survey via the snowballing method (who shared the feedback with the research team).

Here we provide some de-identified responses of participants that were of interest that relate to their inability to answer the survey due to poor or non-existent knowledge on psychedelics.

Quote 1: “I simply cannot respond to a survey about substances that I have no idea about or any sort of knowledge, except that I often heard about cocaine and heroin and I don’t know if they are psychedelics.”

Quote 2: “I would gladly answer the survey questions but I must admit that I haven’t heard about 90% of the listed substances. Likewise, I don’t even know if this substance that is unknown to me is even a psychedelic. And I’ve only been offered the answers Yes and No?! So, I would have to be offered the response option – don’t know.”

Quote 3: “One small comment, without an intention to criticize, but my personal opinion in any case. The topic [of the survey] is interesting and unfortunately currently relevant. However, I gave up on filing the survey until the end, because [my survey] would not faithfully represent my attitude, and even less my knowledge on psychedelics to you. Namely, I am missing the response “don’t know” in most of the survey, because, if I don’t know about something, I cannot agree or disagree. Yet I may agree with the question if it was explained to me what it was about. Otherwise, I am forced to choose either don’t agree or agree for something I don’t actually know about, and that completely changes the whole result of the survey.”
