# Supplementary material for: Validation of a new instrument for assessing attitudes on psychedelics in the general population
Source: Sci Rep. 2022 Oct 29;12:18225. doi: 10.1038/s41598-022-23056-5 (PMC9617880; doi:10.1038/s41598-022-23056-5)
Supplement: Supplementary file 3 — Supplementary Information 3. [file 41598_2022_23056_MOESM3_ESM.docx]

**Appendix C: Description of all statistical indices, tests, and model evaluation procedures used during questionnaire validation**

**Construct validity**

**Supplementary Table C.1.** Descriptions of all model fit indices we used to describe model fit of each structural model assessed during confirmatory factor analysis.

| **Model fit index** | **Category** | **Description** | **Interpretation** |
| --- | --- | --- | --- |
| **Root Mean Square Error of Approximation (RMSEA)** | Parsimony correction index | Incorporates a penalty function for poor model parsimony (i.e. number of freely estimated parameters as expressed by model degrees of freedom (*df*). Also called the *error of approximation index* because it assesses the extent to which a model fits reasonably well in the population (instead of assessing whether the model holds exactly in the population). Insensitive to sample size, sensitive to the number of model parameters. Range is above 0. Upper range is unbounded, but RMSEA values rarely go above 1. | RMSEA values of 0 indicate perfect fit, while values closer to 1 imply good model fit. It is recommended to include the 95% confidence interval when reporting and interpreting the RMSEA. |
| **Standardized Root Mean Squared Residual (SRMR)** | Absolute fit index | Evaluates how well the model estimates reproduce sample variances and covariances. Range of values 0-1. | Values closer to 0 imply good model fit. |
| **Comparative Fit index (CFI)** | Comparative fit index | Evaluates the fit of a user-specified solution in relation to a more restricted, nested baseline model. Range of values 0-1. | Values closer to 1 imply good model fit. |
| **Tucker-Lewis Index (TLI)** | Comparative fit index | Evaluates the fit of a user-specified solution in relation to a more restricted, nested baseline model. Sometimes referred to as the *non-normed fit index*, because its values can fall outside the range of 0-1. Includes a penalty function for adding freely estimated parameters that do not markedly improve the fit of the model. | Values closer to 1 imply good model fit. |
| **Chi-square (χ^2^)** | Absolute fit index | Evaluates how well the model estimates reproduce sample variances and covariances according to a very stringent standard (tests whether the model holds exactly in the population). Large N solutions are often rejected on the basis of the χ^2^ test (a large sample size inflates its values), therefore it is rarely used alone. Often used for nested model comparisons. Degrees of freedom (df) are shown in relation to the χ^2^ value and refer to the number of values that have the freedom to vary in the data sample. | We used χ^2^/df to compare nested models (see *Model evaluation* below). |

There is no clear consensus on which indices should be used to assess goodness of model fit, or which cut-off criteria are most appropriate in a confirmatory factor analysis (Brown, 2015). According to what was described by Brown et al. (Brown, 2015), we chose to follow the cut-off criteria recommended by Hu and Bentler (Hu, 1999). These are described in the *Statistical analysis* section of our manuscript.

**Model evaluation**

We used the likelihood ratio test (Δχ^2^/Δdf, also called the *χ^2^ difference test*) as a method of comparing between nested models, meaning those that have the same amount of items and differ in the number of factors i.e. latent variables (Brown, 2015). The hypothesized 4-factor model was the parent model to which all the other nested models were compared. At df=1, the critical value of χ^2^ is 3.84 (α=.05), so a χ^2^ difference of >3.84 for a change in 1df from parent to the (compared) nested model indicates that the parent model provides a significantly better fit (Brown, 2015).

We also calculated changes in RMSEA (ΔRMSEA) relative to the parent model to compare models more easily.

**References:**

Brown TA. Confirmatory Factor Analysis for Applied Research. 2nd ed. Little TD, editor. New York: Guildord Press; 2015.

Hu L, Bentler PM. Cutoff criteria for fit indexes in covariance structure analysis: Conventional criteria versus new alternatives. Structural Equation Modeling: A Multidisciplinary Journal. 1999;6(1):1-55.
