# Supplementary material for: Validation of a new instrument for assessing attitudes on psychedelics in the general population
Source: Sci Rep. 2022 Oct 29;12:18225. doi: 10.1038/s41598-022-23056-5 (PMC9617880; doi:10.1038/s41598-022-23056-5)
Supplement: Supplementary file 4 — Supplementary Information 4. [file 41598_2022_23056_MOESM4_ESM.docx]

**Appendix D: Description of all demographic data, knowledge on psychedelics results, and results of linear regression modelling and the survey of health care workers**

**Supplementary Figure D.1**. Flowchart showing the number of participants at each stage in the study.

**

**

**Supplementary Table D.1**. Descriptive analysis of demographic information of all included study participants. N=1153.

| **Variable** | **n (%)** |
| --- | --- |
| **Gender** | |
| Male | 426 (36.9) |
| Female | 716 (62.1) |
| Undisclosed | 11 (1.0) |
| **Completed education level** | |
| No primary school education | 0 (0.0) |
| Primary school education | 8 (0.7) |
| High school | 398 (34.5) |
| Undergraduate studies | 183 (15.9) |
| Graduate studies | 429 (37.2) |
| Postgraduate studies | 51 (4.4) |
| PhD studies | 84 (7.3) |
| **Health care worker N (%)** | |
| Yes | 179 (15.5) |
| No | 974 (84.5) |

**Supplementary Table D.2.** Descriptive analysis of different professions of health care workers who were included in the study. N=179.

| **Type of health care worker** | **n (%)** |
| --- | --- |
| Physician | 108 (60.3) |
| Dentist | 29 (16.2) |
| Nurse | 21 (11.7) |
| Pharmacist | 13 (7.3) |
| Physiotherapist | 7 (3.9) |
| Radiology technician | 1 (0.6) |

**Supplementary Table D.3**. Comparison of demographic data between included and excluded participants. Significant P-values are shown in bold. N=1621. Only excluded participants that provided demographic data (468/514, 91.0%) were included in this analysis.

|  | **Included (N=1153)** | **Excluded (N=468)** |  |
| --- | --- | --- | --- |
|  | **n, %** | | P* |
| **Gender** | | | .277 |
| Male | 426 (36.9) | 171 (36.5) |  |
| Female | 716 (62.1) | 288 (61.5) |  |
| Undisclosed | 11 (1.0) | 9 (1.9) |  |
|  | **n, %** | | P* |
| **Education** | | | **.012** |
| No primary school education | 0 (0.0) | 2 (0.4) |  |
| Primary school education | 8 (0.7) | 4 (0.9) |  |
| High school | 398 (34.5) | 194 (41.5) |  |
| Undergraduate studies | 183 (15.9) | 70 (15.0) |  |
| Graduate studies | 429 (37.2) | 152 (32.3) |  |
| Postgraduate studies | 51 (4.4) | 25 (5.3) |  |
| PhD studies | 84 (7.3) | 21 (4.5) |  |
|  | **Median, IQR, 95% CI** | | P** |
| **Age** | | | .717 |
|  | 31.0 (IQR=25.0-42.0, 95% CI=30.0-32.0) | 30.0 (IQR=24.0-43.0, 95% CI=28.0-32.0) |  |

**Supplementary Table D.3 Legend**: Abbrevations: IQR=Interquartile range, CI=confidence interval.
*Chi-square test. Post-hoc analysis showed that there was no significant difference for any education levels between the two groups.
**Mann-Whitney test.

**Supplementary Table D.4**. Descriptive analysis of participants’ responses on the knowledge on psychedelics test. Substances are listed in decreasing frequency of participants who identified them as a psychedelic per each substance group. N=1153.

| **Substance** | **Response, n (%)** | |
| --- | --- | --- |
| **Psychedelics** | **Correctly identified as a psychedelic** | **Incorrectly identified as non-psychedelic** |
| Lysergic acid diethylamide (LSD) | 1038 (90.03) | 115 (9.97) |
| MDMA (ecstasy) | 866 (75.11) | 287 (24.89) |
| Psilocybin | 829 (71.90) | 324 (28.10) |
| DMT | 796 (69.04) | 357 (30.96) |
| Mescaline | 736 (63.83) | 417 (36.17) |
| Peyote | 672 (58.28) | 481 (41.72) |
| Ibogaine | 431 (37.38) | 722 (62.62) |
| **Non-psychedelics** | **Incorrectly identified as a psychedelic** | **Correctly identified as a non-psychedelic** |
| Opium | 690 (59.84) | 463 (40.16) |
| Methamphetamine | 665 (57.68) | 488 (42.32) |
| Heroin | 543 (47.09) | 610 (52.91) |
| Cocaine | 512 (44.41) | 641 (55.59) |
| Dextroamphetamine | 510 (44.23) | 643 (55.77) |
| Ketamine | 454 (39.38) | 699 (60.62) |
| Gamma-hydroxybutyrate (GHB) | 428 (37.12) | 725 (62.88) |
| Rohypnol | 339 (29.40) | 814 (70.60) |
| Oxycodone | 315 (27.32) | 838 (72.68) |
| Haloperidol | 313 (27.15) | 840 (72.85) |
| Mexazolam | 307 (26.63) | 846(73.37) |
| Phenobarbital | 282 (24.46) | 871 (75.54) |
| Modafinil | 244 (21.16) | 909 (78.84) |
| Imipramine | 178 (15.44) | 975 (84.56) |
| Digoxin | 155 (13.44) | 998 (86.56) |

**Supplementary Table D.5**. Results of linear regression modelling used to explore the association of demographic factors with the total APQ score and the scores of all sub-scales. Results with significant p-values are shown in bold. N=1153.

|  | **Predictor variables (β, p-value)** | | | | R^2*^ |
| --- | --- | --- | --- | --- | --- |
| **Outcome variables** | Age | Gender | Education level | HCW status (yes/no) |  |
| Total APQ score | **-0.218, p<.001** | **-0.171, p<.001** | **-0.124, p<.001** | -0.049, p=.091 | 0.126 |
| *Legal use of Psychedelics* score | **-0.211, p<.001** | **-0.159, p<.001** | **-0.072, p=0.027** | **-0.075, p=0.011** | 0.102 |
| *Effects of Psychedelics* score | **-0.204, p<.001** | **-0.199, p<.001** | **-0.117, p<.001** | **-0.061, p=0.036** | 0.130 |
| *Risk Assessment of Psychedelics* score | **0.131, p<.001** | **-0.164, p<.001** | **-0.145, p<.001** | -0.023, 0.444 | 0.089 |
| *Openness to Psychedelics* score | **-0.233, p<.001** | **-0.079, p=0.005** | **-0.108, p=0.001** | -0.014, p=.641 | 0.097 |

**Supplementary Table D.5 Legend:** *R-squared shows the percentage of score variance explained by statistically significant predictor variables.
Abbreviations: APQ=Attitudes on Psychedelics Questionnaire, β=Standardized regression coefficient, R^2^= Coefficient of determination.

**Supplementary Table D.6**. Comparison of scores in knowledge and attitudes on psychedelics between HCW and non-HCW participants. Significant P-values are shown in bold. N=1153.

|  | **Median, IQR, 95% CI** | | P* |
| --- | --- | --- | --- |
| **Variable (theoretical range)** | **Non-HCW (n=974)** | **HCW (n=179)** |  |
| Total APQ score (20.0-100.0) | 66.0 (IQR=56.0-78.0, 95% CI=65.0-67.1) | 62.0 (IQR=53.0-71.8, 95% CI=60.0-64.0) | **<.001** |
| Knowledge test score (0.0-100.0) | 65.9 (IQR=50.0-81.8, 95% CI=63.6-68.2) | 63.6 (IQR=50.0-81.8, 95% CI=59.1-72.7) | .711 |

**Supplementary Table D.6 Legend:** Abbreviations: APQ=Attitudes on Psychedelics Questionnaire, HCW=health care worker.
**Mann-Whitney test.

**Supplementary Table D.7**. Descriptive analysis of health care workers’ responses to the additional set of questions for their sub-group. N=179. Negatively worded items that are reversely coded are marked by (R). For negatively worded items, interpretation of response scores is as follows: Completely agree=1, Agree=2, Neither agree nor disagree=3, Disagree=4, Completely disagree=5. For non-reversed/positive items, the scoring is: Completely disagree=1, Disagree=2, Neither agree nor disagree=3, Agree=4, Completely agree=5.

| **Item text** | **Median score (95%CI)** | **IQR** |
| --- | --- | --- |
| I would be worried if my institution took part in research with psychedelics. **(R)** | 4.0 (4.0-5.0) | 3.0-5.0 |
| I would feel uncomforSupplementary Table recommending psychedelic treatment to a patient, even if there is evidence for its effectiveness and safety. **(R)** | 4.0 (3.0-4.0) | 3.0-4.0 |
| As a health professional, I would be interested in witnessing a psychedelic session with a patient. | 4.0 (4.0-4.0) | 3.0-5.0 |
| If I knew that a psychiatrist used psychedelics, I would be less likely to refer my patient to them. **(R)** | 3.0 (2.0-3.0) | 2.0-4.0 |
| If I knew a psychiatrist who supported the legalization of psychedelics, I would be less likely to refer my patient to them. **(R)** | 3.0 (3.0-4.0) | 2.0-5.0 |

**Supplementary Table D.7 Legend**: CI=Confidence interval, IQR=interquartile range.
