# Supplementary material for: Validation of a new instrument for assessing attitudes on psychedelics in the general population
Source: Sci Rep. 2022 Oct 29;12:18225. doi: 10.1038/s41598-022-23056-5 (PMC9617880; doi:10.1038/s41598-022-23056-5)
Supplement: Supplementary file 5 — Supplementary Information 5. [file 41598_2022_23056_MOESM5_ESM.docx]

**Appendix E: Reliability analysis of the APQ and the psychometric properties of the modified Barnett et al. questionnaire**

**Supplementary Table E.1**. Reliability analysis of the hypothesized 4-factor model with sub-scale ω values if an item is removed and correlations of each item with total of all other items in the sub-scale. Values in bold indicate items those where overall reliability rises if the item is dropped. N=1153. Negatively worded items that are reversely coded are marked by (R).

| **Subscale reliability (McDonald’s ω, 95%CI)** | **Item no.** | **Item text** | **McDonald’s ω if item dropped** | **Item-rest correlation** |
| --- | --- | --- | --- | --- |
| *Legal Use of Psychedelics* (ω=0.842, 95%CI=0.828-0.856) | 1 | Legalizing psychedelics would benefit public health. | 0.786 | 0.708 |
|  | 2 | Those who want to legalize psychedelics have a hidden agenda behind their actions. **(R)** | 0.829 | 0.570 |
|  | 3 | The use of psychedelics for justified medical reasons should be legal. | 0.802 | 0.674 |
|  | 4 | Administering psychedelics to psychiatric patients is safe as long as the treatment conditions are carefully controlled. | 0.825 | 0.573 |
|  | 5 | Administering psychedelics to patients will eventually lead to bad outcomes. **(R)** | 0.804 | 0.674 |
| *Effects of Psychedelics* (ω=0.881, 95%CI=0.870-0.892) | 6 | Psychedelic use is linked to creativity. | 0.866 | 0.657 |
|  | 7 | If more people used psychedelics, the world would be a better place. | 0.838 | 0.783 |
|  | 8 | Recreational use of psychedelics has no practical benefit. **(R)** | 0.866 | 0.665 |
|  | 9 | I am afraid of the effects of psychedelics on physical health. **(R)** | 0.865 | 0.673 |
|  | 10 | Psychedelics can provide valuable spiritual experiences. | 0.842 | 0.772 |
| *Risk Assessment of Psychedelics* (ω=0.841, 95%CI=0.826-0.855) | 11 | Using psychedelics is safe. | 0.787 | 0.725 |
|  | 12 | The use of psychedelics can damage the nervous system. **(R)** | 0.802 | 0.689 |
|  | 13 | Psychedelics are less dangerous than other illegal drugs. | 0.811 | 0.669 |
|  | 14 | A wider use of psychedelics would cause an increase in mental problems. **(R)** | **0.846** | 0.640 |
|  | 15 | Administering psychedelics to patients is not problematic as long as it is performed by a professional. | **0.846** | 0.472 |
| *Openness to Psychedelics* (ω=0.843, 95%CI=0.829-0.858) | 16 | I am optimistic about psychedelic research. | 0.813 | 0.641 |
|  | 17 | I would not agree to use psychedelics for mental health purposes. **(R)** | 0.841 | 0.578 |
|  | 18 | If psychedelic-assisted psychotherapy enters into regular practice, I would be interested in learning more about it. | 0.810 | 0.666 |
|  | 19 | I would be interested in learning about other people’s experiences with psychedelics. | 0.803 | 0.693 |
|  | 20 | I don’t think that learning about psychedelics is worth my time. **(R)** | 0.795 | 0.700 |

**Supplementary Table E.2.** Results of exploratory factor analysis for 7 items from the Barnett et al. questionnaire. Factor loadings over 0.3 are shown in bold. N=1153. Applied rotation method is oblimin. Negatively worded items that are reversely coded are marked by (R).

| **Item no.** | **Item text** | **Factor 1** |
| --- | --- | --- |
| 1 | The use of psychedelics increases the risk for subsequent psychiatric disorders. **(R)** | **0.654** |
| 2 | The use of psychedelics increases the risk for long-term cognitive impairment. **(R)** | **0.736** |
| 3 | The use of psychedelics should be illegal for recreational purposes. **(R)** | **0.466** |
| 4 | The use of psychedelics is unsafe even under medical supervision. **(R)** | **0.790** |
| 5 | The use of psychedelics shows promise in treating psychiatric disorders. | **0.796** |
| 6 | The use of psychedelics may improve outcomes when used during psychotherapy. | **0.799** |
| 7 | The use of psychedelics deserves future research for the treatment of psychiatric disorders. | **0.707** |

**Supplementary Table E.3**. Reliability analysis of the Barnett et al. questionnaire with scale ω value if an item is removed and correlations of each item with total of all other items in the sub-scale. Values in bold indicate items those where overall reliability rises if the item is dropped. N=1153. Negatively worded items that are reversely coded are marked by (R).

| **Total scale reliability (McDonald’s ω, 95%CI)** | **Item no.** | **Item text** | **McDonald’s ω if item dropped** | **Item-rest correlation** |
| --- | --- | --- | --- | --- |
| (ω=0.863, 95%CI=0.851-0.875) | 1 | The use of psychedelics increases the risk for subsequent psychiatric disorders. **(R)** | 0.846 | 0.614 |
|  | 2 | The use of psychedelics increases the risk for long-term cognitive impairment. **(R)** | 0.837 | 0.690 |
|  | **3** | The use of psychedelics should be illegal for recreational purposes. **(R)** | **0.883** | 0.441 |
|  | 4 | The use of psychedelics is unsafe even under medical supervision. **(R)** | 0.829 | 0.724 |
|  | 5 | The use of psychedelics shows promise in treating psychiatric disorders. | 0.837 | 0.716 |
|  | 6 | The use of psychedelics may improve outcomes when used during psychotherapy. | 0.835 | 0.720 |
|  | 7 | The use of psychedelics deserves future research for the treatment of psychiatric disorders. | 0.845 | 0.639 |

**Supplementary Table E.3 Legend:** Abbreviations: CI=confidence interval.

**Supplementary Table E.4**. Model fit indices for two assessed structural models of the Barnett et al. questionnaire. The model with the best fit is shown in bold. N=1153. The modified version of the questionnaire has item 3 removed and a total of 6 items.

|  | **RMSEA (95%CI)** | **SRMR** | **CFI** | **TLI** | **χ^2^ (df)** | **ΔRMSEA**** |
| --- | --- | --- | --- | --- | --- | --- |
| **Model** |  |  |  |  |  |  |
| Modified Barnett et al. questionnaire | 0.081 (0.064-0.100) | 0.065 | 0.984 | 0.970 | 69.01 (8) | - |
| **Barnett et al. questionnaire** | **0.063 (0.050-0.078)** | **0.058** | **0.987** | **0.978** | **73.37 (13)** | **-0.018** |

**Supplementary Table E.*5.*** Factor loading estimates of all APQ items and their factors.

| **Factor** | **Item** | **Estimate** | **Standard Error** | **Std. Est (lv)*** | **Std. Est (all)** |
| --- | --- | --- | --- | --- | --- |
| *Legal Use of Psychedelics* | Q1 | 0.977 | 0.016 | 0.977 | 0.837 |
|  | Q2 | 0.664 | 0.013 | 0.664 | 0.647 |
|  | Q3 | 0.705 | 0.013 | 0.705 | 0.708 |
|  | Q4 | 0.544 | 0.011 | 0.544 | 0.595 |
|  | Q5 | 0.729 | 0.013 | 0.729 | 0.758 |
| *Effects of Psychedelics* | Q6 | 0.761 | 0.013 | 0.761 | 0.660 |
|  | Q7 | 1.069 | 0.015 | 1.069 | 0.849 |
|  | Q8 | 0.846 | 0.014 | 0.846 | 0.717 |
|  | Q9 | 0.993 | 0.015 | 0.993 | 0.783 |
|  | Q10 | 1.060 | 0.015 | 1.060 | 0.826 |
| *Risk Assessment of Psychedelics* | Q11 | 0.739 | 0.013 | 0.739 | 0.760 |
|  | Q12 | 0.726 | 0.013 | 0.726 | 0.711 |
|  | Q13 | 0.835 | 0.015 | 0.835 | 0.725 |
|  | Q14 | 0.796 | 0.014 | 0.796 | 0.762 |
|  | Q15 | 0.589 | 0.012 | 0.589 | 0.595 |
| *Openness to Psychedelics* | Q16 | 0.679 | 0.013 | 0.679 | 0.726 |
|  | Q17 | 1.005 | 0.017 | 1.005 | 0.829 |
|  | Q18 | 0.564 | 0.013 | 0.564 | 0.614 |
|  | Q19 | 0.634 | 0.014 | 0.634 | 0.636 |
|  | Q20 | 0.743 | 0.014 | 0.743 | 0.717 |

**Supplementary Table E.5 Legend:** *Standardized estimate, only latent variables standardized.

**Standardized estimate, all variables standardized.
