# Supplementary material for: Validation of a new instrument for assessing attitudes on psychedelics in the general population
Source: Sci Rep. 2022 Oct 29;12:18225. doi: 10.1038/s41598-022-23056-5 (PMC9617880; doi:10.1038/s41598-022-23056-5)
Supplement: Supplementary file 6 — Supplementary Information 6. [file 41598_2022_23056_MOESM6_ESM.docx]

**Appendix F: Items from the Attitudes on Psychedelics Questionnaire (APQ) in Croatian**

**Supplementary Table F.1.** The hypothesized model of the APQ in Croatian. Negatively worded items that are reversely coded are marked by (R).

|  | **Item no.** | **Item text** |
| --- | --- | --- |
| **Sub-scale** |  |  |
| *Legalna uporaba psihodelika* | 1 | Legalizacija psihodelika pridonijela bi javnom zdravstvu. |
|  | 2 | Osobe koje žele legalizirati psihodelike imaju skriveni plan u pozadini svojih postupaka. **(R)** |
|  | 3 | Uporaba psihodelika iz opravdanih medicinskih razloga trebala bi biti legalna. |
|  | 4 | Davanje psihodelika psihijatrijskim bolesnicima sigurno je dok su god uvjeti liječenja pažljivo kontrolirani. |
|  | 5 | Davanje psihodelika pacijentima s vremenom dovodi do loših ishoda. **(R)** |
| *Učinci psihodelika* | 6 | Korištenje psihodelika povezano je s kreativnošću. |
|  | 7 | Kada bi više ljudi koristilo psihodelike, svijet bi bio bolje mjesto. |
|  | 8 | Rekreacijsko korištenje psihodelika nema praktičnu korist. **(R)** |
|  | 9 | Strah me učinaka psihodelika na tjelesno zdravlje. **(R)** |
|  | 10 | Psihodelici mogu pružiti vrijedna duhovna iskustva. |
| *Procjena rizika psihodelika* | 11 | Uporaba psihodelika je sigurna. |
|  | 12 | Uporaba psihodelika može oštetiti živčani sustav. **(R)** |
|  | 13 | Psihodelici su manje opasni od ostalih ilegalnih droga. |
|  | 14 | Šira uporaba psihodelika dovela bi do povećanja broja mentalnih problema. **(R)** |
|  | 15 | Davanje psihodelika pacijentima nije problematično dok god to radi stručna osoba. |
| *Otvorenost psihodelicima* | 16 | Optimističan/na sam oko znanstvenih istraživanja o psihodelicima. |
|  | 17 | Ne bih pristao/la koristiti psihodelike u svrhe vezane za mentalno zdravlje. **(R)** |
|  | 18 | Da psihoterapija potpomognuta psihodelicima uđe u svakodnevnu praksu, zanimalo bi me doznati više o njima. |
|  | 19 | Bio bih zainteresiran/na za učenje o iskustvima drugih ljudi s psihodelicima. |
|  | 20 | Mislim da upoznavanje s psihodelicima nije vrijedno moga vremena. **(R)** |
